# Early interventions in mental health services: a rapid review of systematic review

**DOI:** 10.3389/fpubh.2026.1782381

**Published:** 2026-09-03

**Authors:** Rosina Anna Adu, Medard Kofi Adu, Belinda Agyapong, Vincent I. O. Agyapong

**Affiliations:** 1 Department of Psychiatry, Faculty of Medicine, Dalhousie University, Halifax, NS, Canada; 2 Mental Health and Addictions Program, Nova Scotia Health, Halifax, NS, Canada

**Keywords:** cost-effectiveness, early intervention, early psychosis, mental health services, service accessibility, systematic reviews, youth mental health

## Abstract

**Background:**

Mental health disorders account for a substantial global burden of disease, with many individuals experiencing significant delays in receiving appropriate care. Early intervention has emerged as a key strategy to improve clinical, functional, and economic outcomes across the mental health continuum. Although evidence surrounding early intervention is well established, a broader synthesis of early intervention models across diagnoses, age groups, and service settings has been lacking.

**Objectives:**

This Rapid Review sought to map and synthesize systematic review evidence on early intervention mental health services to inform policy, planning, and implementation strategies.

**Methods:**

This rapid review of systematic reviews and meta-analyses was conducted to provide a timely synthesis of high-level evidence on early intervention mental health services. Seven electronic databases (MEDLINE, PubMed, Embase, PsycINFO, CINAHL, Cochrane Library, and Scopus) were searched for English-language systematic reviews and meta-analyses published between January 2015 and June 2025. Eligible reviews examined early intervention models for individuals with or at risk of mental health disorders and reported outcomes related to effectiveness, accessibility, cost-effectiveness, patient satisfaction, or system-level performance. Data were synthesized narratively.

**Results:**

Eleven systematic reviews and meta-analyses met the inclusion criteria. Early Intervention in Psychosis services consistently reduced hospitalization, treatment discontinuation, and symptom severity while improving social and vocational functioning. Family-focused interventions enhanced caregiver knowledge and wellbeing. Brief crisis intervention models for children and youth reduced emergency department revisits and inpatient admissions. Digital and smartphone-enabled interventions demonstrated feasibility and user engagement, though evidence for sustained clinical benefit remains limited. Economic evaluations showed early psychosis intervention to be cost-effective relative to standard care, but data from low- and middle-income countries were sparse. Key barriers to effective implementation included stigma, fragmented care pathways, and insufficient mental health literacy; facilitators included family involvement, continuity of care, and flexible service delivery.

**Conclusions:**

Early intervention models improve outcomes and are often cost-efficient. To maximize impact, health systems should prioritize accessibility, continuity, family engagement, and cultural responsiveness, while expanding evaluation of digital and community-based approaches across diverse settings.

## Introduction

Mental health disorders represent the single most significant contributor to the global burden of disease, surpassing all other health conditions in their overall impact: they account for approximately 13% of the global burden of disease, underscoring their significant impact on individuals, families, and societies (1). When examined in terms of specific health metrics, mental illnesses are responsible for 32.4% of all years lived with disability (YLDs), reflecting the prolonged and often chronic nature of these conditions that can impair daily functioning over a lifetime (2). In addition, they make up 13% of disability-adjusted life years (DALYs), a comprehensive measure that combines years lost due to premature death and years lived with disability (2). This growing burden underscores not only the scale of mental health challenges globally but also the urgency of addressing mental health needs through comprehensive and sustainable strategies. Despite the increasing recognition of the importance of mental health, services in many regions remain underfunded, stigmatized, and fragmented, requiring innovative and multifaceted approaches to overcome these barriers (3–5). This highlights the urgent need for prevention, early intervention, and accessible treatment to reduce their impact.

Addressing the unmet need for high-quality mental health services that are responsive to patients' needs and uphold citizens' right to mental health can be achieved gradually through strategic planning, regular annual evaluations, and targeted improvements in service quality (6). Furthermore, both qualitative and quantitative assessments using well-defined quality indicators are essential for identifying the needs of disadvantaged social groups. Such evaluations enable the state to take informed political action to guarantee universal and equitable access to mental health services for all (6).

The quality of mental health services varies greatly both across and within countries, often reflected in the gap within each health system between the clinical guidelines established by international scientific bodies and the realities of daily practice (7). Since psychiatric systems differ from one country to another, the clinical decisions made for the same symptoms or conditions can vary, influenced by resource availability and opportunities to deliver quality care. Research assessing intervention quality consistently shows that everyday clinical practice generally falls short of the standards outlined in national and international guidelines (7).

### Early interventions in mental health services

Preventive mental health strategies are typically categorized into three levels: universal prevention, which targets the general population; selective prevention, which focuses on individuals at higher risk; and indicated prevention, which is aimed at those already showing early signs of a mental health issue (8). Indicated prevention often involves timely interventions in primary and community mental health settings, directed at individuals exhibiting early symptoms (9).

Early intervention, a well-established concept in healthcare services, emphasizes the timely identification and implementation of stage-specific treatments (10). When treatment is provided proactively and tailored to the stage of illness, it can prevent or mitigate adverse outcomes, thereby reducing the future need for expensive or invasive care (11). However, despite its proven benefits, the adoption of early intervention strategies within mental health has been relatively slow (12). Most mental illnesses emerge during adolescence and early adulthood (approximately ages 12–30), a critical period of social, psychological, and biological development (13, 14). Delays in receiving effective care during this phase can disrupt essential developmental milestones and have enduring impacts on individuals' health, social relationships, and occupational outcomes (15).

Service delivery for mental health care often fails to align with the patterns of onset and disease burden associated with mental disorders, even within advanced health systems (1). Globally, access to evidence-based mental health services remains inadequate, and for those who do eventually receive care, it typically occurs only after significant delays (5, 13, 16). The duration of untreated illness, thus the time between the onset of a psychiatric disorder and the start of appropriate treatment, can span from 1–2 years in cases of psychosis to as long as 10 years for obsessive compulsive disorder (OCD) (17–19). Over time, untreated mental illnesses may become more deeply rooted due to factors such as functional decline, neurobiological changes, and entrenched behavioral patterns (20, 21). A prolonged duration of untreated illness has been consistently linked with poorer symptom improvement, diminished functioning, and reduced treatment responsiveness across diagnostic categories (15, 20).

The mental health field has made notable progress in developing early intervention frameworks, expanding early psychosis services and creating youth-focused programs. Evidence shows that early intervention effectively prevents or delays mental disorders and, when systematically implemented, is both accessible and cost-effective (16, 22).

While early psychosis programs are now well established (16, 23), there is a further need for future efforts to focus on maintaining fidelity, ensuring continuity, and extending early intervention to a broader range of severe and non-psychotic disorders. More high-quality studies are needed to determine the most effective intervention intensity, duration, and strategies for improving functional outcomes. Sustaining treatment gains is essential, premature disengagement risks relapse and inconsistent care. Stage-specific, proportionately resourced interventions, similar to models in cancer care, offer the best means to preserve recovery and long-term stability (24).

Efforts to advance early intervention, especially for subthreshold psychosis and reform youth mental health services, have sparked debate. Critics worry that expanding mental healthcare may lead to overdiagnosis and overtreatment, or that limited resources should focus on severe, late-stage illnesses (16). However, investing in early detection can ultimately reduce strain on later-stage services, much like in other chronic diseases (25).

Concerns about overtreatment may persist; however, substantial treatment gaps and delays in accessing appropriate mental health care remain widespread (26, 27). While premature medication use occasionally occurs, this usually reflects insufficient psychosocial support rather than flaws in early intervention (16, 26). Questions about false positives in identifying at-risk individuals remain, with roughly 36% developing psychosis within 3 years (28). Nevertheless, prediction accuracy improves in targeted high-risk populations (29). Stepped-care models help minimize overtreatment by initially providing the least intensive evidence-based interventions, including psychoeducation, cognitive behavioral therapy, family support, and close clinical monitoring, while reserving pharmacological treatment for individuals whose symptoms persist, worsen, or progress to later clinical stages (22). Limited access to psychosocial services may otherwise result in premature reliance on medication. Similarly, the transdiagnostic clinical staging model conceptualizes mental disorders as progressing through successive stages, allowing interventions to be matched to illness severity rather than diagnostic category (16, 22). This approach reduces unnecessary treatment exposure, minimizes stigma, and promotes safer, more effective early intervention.

The evidence strongly supports widespread adoption of early intervention, especially for psychosis (23). Further progress depends on addressing subthreshold cases, reducing untreated durations, maintaining model fidelity, and ensuring continuity of care. While psychosis research leads the field, evidence for non-psychotic disorders including major depressive disorder, bipolar disorder, anxiety disorders, and eating disorders is growing (30, 31). Early intervention for bipolar disorder shows strong potential, as treatment is most effective early and relapse can cause brain changes (32). Similarly, for depression, early intervention remains the optimal approach and may even prevent full disorder onset (31).

Preventive healthcare strategies, particularly early intervention, enhance treatment effectiveness, reduce the pain and damage caused by diseases, and improve overall quality of life for families. They focus on health promotion, early screening, and rehabilitation, ultimately restoring maximum function and minimizing disability (33).

While the evidence base for early intervention is growing, existing systematic reviews tend to focus on specific diagnoses (e.g., psychosis, depression), subpopulations (e.g., youth), or single outcome measures (20, 23, 24). There is a lack of a comprehensive synthesis that addresses multiple outcomes of interest across the overall population, broadly defined (i.e., all age groups). Furthermore, the landscape of early intervention services has undergone significant evolution in recent years, driven by technological advancements, service reforms, and pandemic-related disruptions (34, 35).

This rapid review aims to address this gap by synthesizing evidence from systematic reviews on early intervention mental health services across all age groups, with a focus on five key outcome domains: service effectiveness, accessibility, cost-effectiveness, patient satisfaction, and system-level outcomes. In this review, service effectiveness refers to clinical and functional outcomes; accessibility reflected service availability, timely access, uptake, and engagement; cost-effectiveness considered costs in relation to outcomes and resource use; patient satisfaction captured experiences and acceptability of care; and system-level outcomes included healthcare utilization, service coordination, and overall service performance. The findings will inform policymakers, service planners, and clinicians seeking to improve mental health service delivery across diverse settings.

### Research questions

1. What is the extent of the literature on early intervention services for mental health disorders?
2. What are the types and characteristics of early intervention services and care pathways reported in the literature?
3. What evidence exists regarding the service effectiveness, accessibility, cost-effectiveness, patient satisfaction, and system-level outcomes of early intervention services?
4. What factors influence the implementation of early intervention services (i.e., barriers and facilitators to implementation)?

## Methods

This rapid review of systematic reviews and meta-analyses was conducted to provide a timely synthesis of high-level evidence on early intervention mental health services. The review adopted a streamlined evidence synthesis approach informed by established rapid review methodologies and principles of systematic review conduct. The review question and eligibility criteria were developed using the Population, Intervention, Comparator, and Outcome (PICO) framework (36). Reporting of the review process was guided by the Preferred Reporting Items for Systematic Reviews and Meta-Analyses (PRISMA 2020) Statement (37). A rapid review framework was selected to enable a pragmatic and timely synthesis of higher-level evidence relevant to mental health policy, service planning, and implementation. This approach allowed for streamlined evidence synthesis while maintaining systematic and transparent review procedures.

### Data search

A comprehensive search strategy was applied to seven electronic databases: MEDLINE, PubMed, Embase, PsycINFO, CINAHL, Cochrane Library, and Scopus. The search used a combination of controlled vocabulary terms (e.g., MeSH) and free-text keywords relating to early intervention and mental health services, with Boolean operators and truncation applied as appropriate. Reference lists of included reviews and related publications were also screened to identify additional eligible studies. To capture contemporary evidence, the search was restricted to English-language publications from January 2015 to June 2025. The search were conducted in June 2025. For consistency, the literature search was conducted by one study team member (R. A.), while all retrieved results were independently reviewed by the study team to ensure the accuracy and completeness of the search findings. Database-specific search strategies used to operationalize the concepts of early intervention, mental health services, and review-level evidence synthesis are provided in Supplementary Appendix 1.

### Inclusion criteria

Studies were included if they were meta-analysis, or systematic reviews, with or without meta-analyses, that focused on early intervention strategies or models of care for individuals across the lifespan experiencing or at risk of mental health disorders. Reviews were required to assess at least one of the following outcomes: effectiveness, accessibility, cost-effectiveness, patient satisfaction, or system-level outcomes. Only peer-reviewed articles published in English were considered. Peer-reviewed status was verified during full-text screening using database indexing information and journal publisher records where necessary. Eligible studies were limited to articles published in peer-reviewed academic journals indexed within the selected electronic databases. Reviews were excluded if they were primary studies, narrative reviews, scoping reviews, rapid reviews, umbrella reviews, protocols, dissertations, conference abstracts, or other forms of non-peer-reviewed literature.

### Study selection

All retrieved records were imported into Covidence, a systematic review software management. Duplicate records were automatically identified and removed within Covidence. Titles and abstracts were then screened independently by two reviewers (RAA and MKA) against the eligibility criteria. Full-text articles of potentially relevant studies were retrieved and assessed in detail by two reviewers (RAA and MKA). Discrepancies at any stage were resolved through discussion, by a third reviewer (BA).

### Data extraction

Data extraction was performed using a standardized data extraction form in Microsoft Excel. The form captured bibliographic details such as author, year, and review characteristics including type of review, number of included studies, and databases searched; population characteristics such as age group and diagnosis or risk profile; intervention details including type, mode of delivery, setting, and duration; and reported outcomes relating to effectiveness, accessibility, cost-effectiveness, patient satisfaction, and system-level outcomes. Key findings, conclusions, and identified research gaps were also documented. Data extraction was performed by one reviewer and independently verified by a second reviewer for accuracy and completeness. The characteristics of the included studies are summarized in Table 1.

**Table 1:** Characteristics and key findings of the included systematic reviews.

References/country of origin	Study aims	Number of included primary studies	Study population	Type of early intervention/treatment	Study design/review type	Outcomes evaluated	Risk of bias assessment	Significant findings	Conclusion	Limitations
# 1 Correll et al. (23)/U.S.A	Comparing early intervention services (EIS) with treatment as usual (TAU) for early-phase psychosis.	10	2,176	Early intervention in psychosis (EIP) and early intervention services (EIS), which were compared against treatment as usual	Systematic review and meta-analysis	The coprimary outcomes were all-cause treatment discontinuation and at least 1 psychiatric hospitalization during the treatment period (excluding a potential initial hospitalization before the initiation of the EIS intervention).	Not reported	EIS was associated with better outcomes than TAU at the end of treatment for all 13 meta-analyzable outcomes. These outcomes included the following: all-cause treatment discontinuation [risk ratio (RR), 0.70; 95% CI, 0.61–0.80; P < 0.001], at least 1 psychiatric hospitalization (RR, 0.74; 95% CI, 0.61–0.90; P = 0.003), involvement in school or work (RR, 1.13; 95% CI, 1.03–1.24; P = 0.01), total symptom severity (standardized mean difference [SMD], −0.32; 95% CI, −0.47 to −0.17; *P* < 0.001), positive symptom severity (SMD, −0.22; 95% CI, −0.32 to −0.11; *P* < 0.001), and negative symptom severity (SMD, −0.28; 95% CI, −0.42 to −0.14; *P* < 0.001). Superiority of EIS regarding all outcomes was evident at 6, 9–12, and 18–24 months of treatment (except for general symptom severity and depressive symptom severity at 18–24 months).	In early-phase psychosis, early intervention services (EIS) are superior to treatment as usual (TAU) across all meta-analyzable outcomes. These results support the need for funding and the use of EIS in patients with early-phase psychosis.	The meta-analysis was constrained by a modest number of heterogeneous trials and participants, limiting the ability to assess publication bias, subgroup effects, and the independent impact of specific intervention components.
# 2 Mascayano et al. (41)/U.S.A	To analyze rates of disengagement in early psychosis services and identify predictors of disengagement in these settings.	20	Ranged from 21 to 786	Predictors of service disengagement	Systematic review	The study evaluated disengagement from Early Intervention Services (EIS) for psychosis, which generally meant: No contact with service providers, dropping out of treatment, refusal of care and leaving services despite clinical advice. The study evaluated disengagement from Early Intervention Services (EIS) for psychosis, which generally meant: No contact with service providers, dropping out of treatment, refusal of care and leaving services despite clinical advice.	Not reported	Twenty studies met the inclusion criteria. Disengagement rates (12% to 53%) and definitions of disengagement varied widely across these studies. Most did not find a compelling association between predisposing factors (e.g., age) and disengagement. Enabling factors, such as a lack of family support and living alone, were consistently associated with increased disengagement across studies. Finally, need factors, such as lower medication adherence and higher drug misuse, were associated with a higher risk for disengagement.	Enabling and need factors seemed to be the most predictive of disengagement from early psychosis services. Substantial between-study variation in identified predictors of disengagement may be addressed by developing and applying a consensus definition of disengagement in future research.	This review was limited by inconsistent findings across studies, heterogeneity in research designs and outcome definitions, and potential selection bias, all of which hinder the ability to draw generalizable conclusions or conduct a meta-analysis; moreover, context-specific factors such as health service organization may have further influenced the observed results.
# 3 Sizer et al. (38)/Australia	To identify the literature on the outcomes of people who initially presented via an ARMS clinic and later transitioned to a psychotic disorder (UHR-T), compared to those who presented directly to an EI service with a FEP (FEP-D).	3	49–147	Evaluation of clinical outcomes	Systematic review and meta-analysis	The studies primarily evaluated clinical outcomes, focusing on hospitalization and involuntary treatment rates. Overall, no significant differences were found between groups, except for one study that reported reduced hospitalizations and involuntary treatment among UHR-T individuals.	Not reported	This systematic review has identified that there is limited research in comparing the outcomes of those with a first episode of psychosis who enter services via an ARMS clinic to those who present directly with a FEP. Only three studies were identified, of which only two had the primary objective of comparing the groups; the other study addressed secondary objectives. The majority of studies found no differences in outcomes for UHR-T individuals, except for one study that reported lower rates of hospitalization and involuntary treatment.	The limited research identified in this systematic review found broadly similar outcomes for people with a FEP who presented initially through an ARMS clinic or directly to a FEP service, except for hospitalization. It was found that UHR-T individuals had a follow-up, and one study identified lower rates of involuntary admissions among those who presented via an ARMS clinic. lower risk of hospital admission at	This review is limited by the small number and size of studies, with only two directly comparing the groups and one rated as moderate quality; nonetheless, these gaps highlight the lack of research on this important topic.
# 4 Aceituno et al. (40)/United Kingdom	To review the cost-effective evidence of EIP services worldwide	16	6,597	The cost-effectiveness evidence of Early Intervention Services	Systematic review	This systematic review evaluated the cost-effectiveness of Early Intervention in Psychosis (EIP) services compared with standard care for individuals with first-episode psychosis and those at clinical high risk. Across 16 studies, outcomes such as incremental cost-effectiveness ratios, quality-adjusted life-years (QALYs), and healthcare resource use were assessed. The findings consistently indicated that EIP services are generally more cost-effective and often cost saving compared to usual care, although the overall certainty of the evidence was moderate due to methodological limitations and study heterogeneity	Risk of bias was mixed across trials, mainly due to selection bias and the inherent lack of blinding in the study design. Participant blinding was absent, and investigator/analyst masking was unclear in over half the studies. For economic evaluations, nearly 60% met most CHEC quality criteria, while 21% failed to meet half. Key limitations included limited perspectives (only 43.7% considered costs beyond healthcare) and incomplete use of incremental analyses and discounting.	The overall evidence was consistent in the cost-effectiveness of EIP compared with standard care for the first episode of psychosis and the Clinical High Risk for Psychosis paradigm. Such evidence was replicated across different health systems, primarily in high-income countries. The methodological quality of such evidence, however, was moderate, and heterogeneity was significant across the studies.	There is consistent evidence that implementing EIP services may be a cost-effective alternative across different health systems. Such evidence, nevertheless, derives from heterogeneous, sometimes methodologically flawed studies, reducing the certainty of the statement. More effort must be made to rigorously assess the value of this intervention before expanding it across systems with more constrained mental health budgets.	This systematic review has several limitations. For instance, despite the comprehensiveness of the search, unpublished or local economic evaluations could be missed. Furthermore, given the high heterogeneity across studies, a meta-analysis was not feasible. Finally, as discussed, this systematic review included only studies conducted in high-income countries, limiting its generalizability to settings with fewer resources.
# 5 Day and Petrakis (42)/Australia	The objective of this study was to explore the development of family intervention in an early psychosis context. The role played by family members and friends in the recovery of individuals with early psychosis is extremely important, and there is a growing body of literature that reflects this. However, how mental health services can best support and utilize family and friends as a core component in recovery from early psychosis is not yet established.	14	164	Family and caregiver interventions	Systematic review	The study evaluated the effectiveness of family and caregiver interventions delivered within Early Intervention in Psychosis (EIP) services on outcomes for people with psychosis focusing on symptom severity, relapse rates hospitalization/readmission rates, social and occupational functioning, medication adherence, overall recovery, and quality of life. It also examined the impact of these interventions on families and caregivers in relation to caregiver burden, stress levels, coping ability, knowledge, understanding of psychosis, communication and problem-solving skills.	Not reported Not reported	The review indicates that local practice-based studies show group interventions can enhance knowledge about psychosis, support carers‘ wellbeing, and expand family involvement in recovery. While these approaches generally show benefits, challenges remain, including stigma, carers' competing responsibilities, and resource demands for implementing supportive programs.	The review shows substantial research, particularly practice-based, on family interventions in early psychosis, though interventions and settings vary widely. Including smaller qualitative studies provides valuable insights into diverse initiatives across early psychosis services, partially addressing questions raised by Askey et al. (58)	The limited number of studies in this important area of mental health services research necessitated a mixed-methods approach and broader inclusion criteria than those of a systematic review. Nevertheless, a robust approach to the search strategy, the databases accessed, and a close reading of articles was important to enable comparisons between findings, studies, and services, and to uncover key learning to share with practitioner colleagues attempting to advance work with families as core business in services.
# 6 Camacho et al. (34)/U.S.A	This study aimed to review the published literature on smartphone technology to enhance care for patients with prodromal and early course psychosis and schizophrenia, and to analyze studies by type, aligned with coordinated specialty care domains.	21	Not reported	Digital and smartphone-enabled interventions	Systematic review	The study evaluated the risk of harm for participants included in the and indicated that there was a low risk of harm from using app-based tools in individuals with prodromal and first-episode schizophrenia, with no reported adverse events. However, some users experienced increased stress related to concerns about digital monitoring, a discomfort also seen in the general population. While certain apps included privacy protections such as fingerprint or PIN access, security measures were not consistently reported across studies.	Not reported	Feasibility studies showed a high user engagement and interest among patients, monitoring studies demonstrated a correlation between app assessments and clinical outcomes, and intervention studies indicated that these apps have the potential to advance care. 18 studies reported on app use for coordinated specialty care case management. No app studies focused on employment and education, coordination with primary care services, family education and support.	Although limited, research on smartphone apps for prodromal and first-episode psychosis is rapidly growing and shows promise for monitoring and interventions. High adoption and feasibility support future development of coordinated care–specific app evaluation tools and scales.	This review has several limitations. Our search strategy may not have captured all relevant studies due to varied terminology across prodromal, first-episode, and smartphone tools. Clinical judgment was required to include certain papers, such as the Crosscheck case report, when populations were not clearly defined. Additionally, unpublished apps or studies may contribute to publication bias. We mitigated these limitations by collaborating with a librarian to develop and execute the search strategy.
# 7 Tiller et al. (43)/United Kingdom (Netherlands)	This systematic review examines barriers and facilitators to accessing these services to identify targets for service-level change.	10	5–24 participants (Qualitative study) 78–200 (Quantitative study)	Barriers and facilitators to service access	Systematic review	This review sought to identify key barriers and facilitators to seeking access to early intervention for psychosis services.	Not reported	The search yielded 10 studies. Mental health stigma and discrimination predict DUP, compounded by structural barriers which limit the impact of early intervention services on timely access to recommended treatments. Synthesis of qualitative studies generated three themes: knowledge, relationships, and stigma. Lack of knowledge, absence of supportive relationships (social and professional), and self-stigma constitute significant barriers to accessing early intervention services.	This is the first review of the barriers and facilitators to seeking access to early intervention services. The findings highlight public health and secondary care service targets to expedite access to recommended treatments and thereby reduce the DUP.	This study did not identify any limitations.
# 8 Eapen et al. (44)/United Kingdom	To synthesize evidence on the effectiveness of brief interventions in improving mental health outcomes for CYP (0–17 years) presenting with an acute mental health condition.	30	Not reported	Brief, crisis-focused approaches targeting children and adolescents	Systematic review	The study evaluated whether brief interventions effectively decreased the reliance on emergency department assessments, reevaluations or in-patient admissions among children and young people experiencing mental health crises.	Not reported	We reviewed 30 studies on brief interventions for children and young people with acute mental health conditions, including crisis intervention, integrated services, therapies, pharmacotherapy, and in-hospital care. Only one high-quality study on crisis intervention showed reduced hospital stays and ED return visits, while moderate-quality studies suggested benefits from multimodal approaches.	This review provides evidence to substantiate the benefits of brief interventions across different settings, reducing the burden of inpatient hospitalizations and emergency department readmissions.	Given the rising number of CYP presenting to emergency departments in crisis, brief interventions may improve outcomes. Systematic evaluation using consistent measures is needed to assess their impact on ED presentations, admissions, LOS, and psychological outcomes for CYP and families. Research should also examine barriers to implementation, compare interventions with appropriate controls, and evaluate their effects on other parts of the mental health system, including private, primary, and community services.
# 9 Gee et al. (46)/United Kingdom	This paper aims to provide a meta-analytic review of randomized controlled trials of indicated psychological interventions for young people aged 10-19 with elevated symptoms of depression and/or anxiety.	45	Not reported	Psychological interventions for young people aged 10–19 with elevated symptoms of depression and/or anxiety.	Meta-analysis	Symptoms of depression and anxiety in adolescents	Not reported	Data from 45 trials were analyzed. Most interventions studied used cognitive and behavioral strategies. Few studies met methodological quality criteria, but the effect size was not associated with study quality. Indicated school-based interventions had a small effect on reducing depression symptoms (SMD = 0.34, 95% CI −0.48, −0.21) and a medium effect on reducing anxiety symptoms (SMD=-0.49, 95% CI −0.79, −0.19) immediately post-intervention. Subgroup analyses indicated that interventions delivered by internal school staff did not have significant effects on symptoms. Reductions in depression were maintained at short-term ( ≤ 6 months) but not medium (>6 months ≤ 12) or long-term (>12 months) follow-up. Reductions in anxiety symptoms were not maintained at any follow-up.	Indicated school-based interventions are effective at reducing symptoms of depression and anxiety in adolescents immediately post-intervention, but there is little evidence that these reductions are maintained. The current evidence base does not support interventions delivered by school staff. Further high-quality randomized controlled trials incorporating assessment of longer-term outcomes are adherence, engagement, and costs would needed to justify increased investment in school-based interventions for adolescent depression and anxiety.	This review has several limitations. Some eligible studies lacked accessible data, introducing potential bias. Planned adjustments for cluster-randomized trials were not feasible due to missing intra-cluster correlations and cluster sizes, which may have affected results despite sensitivity analyses suggesting minimal impact. Future trials should report these data to support reviews. Additionally, evaluating adverse events, further strengthen evidence-based decision-making.
#10 Ly et al. (45)/England	The current service organization is not adapted for youth with or at risk of mental illness. Access, engagement and continuity of care are notorious challenges, particularly during the transition from adolescence to adulthood, when youths are transferred to adult services. An HTA was initiated to evaluate the efficacy of programs for which admission is not a function of the legal age of majority.	5	Not reported	Transition pathways between child and adult mental health services	Systematic review	The outcomes evaluated included Accessibility, continuity, recovery, acceptability, satisfaction, engagement, early intervention, autonomy or suitability	Not reported	From 1,841 references, 5 studies were included, 3 on early intervention (EI) for psychosis. EI alone did not reduce the duration of untreated psychosis, but multifaceted campaigns were more effective. EI appeared to reduce hospitalizations, and analysis of service user and professional experiences highlighted factors influencing implementation, including interagency collaboration and person-centered care.	Healthcare policies need to support further research and development of EI, where admission is not determined by the legal age of majority or diagnostic criteria, particularly for youths at risk.	Not reported
# 11 Williams et al. (39)/England	To identify studies by comparing interventions for early psychosis. We performed a series of component network meta-analyses (cNMA) to determine which components of the interventions provided by EIP services are associated with sustained reduction of psychotic symptoms and improved acceptability	37	4,599	Early intervention in psychosis (EIP) and early intervention services (EIS), which were compared against treatment as usual	Systematic review and meta-analysis	Outcome evaluated ere. (i) change in severity of positive and negative psychotic symptoms as measured on a validated scale at 3-month and 1-year follow-up (after the initiation of the intervention), and (ii) discontinuation from treatment due to any reason (which we note may have included recovery and is not necessarily indicative of a poor outcome). Our secondary outcomes were (i) severity of depressive symptoms and (ii) social functioning assessed using a continuous validated rating scale at 1-year follow-up.	Two researchers independently assessed the risk of bias for negative symptoms at 3-month and 1-year follow-up using the Risk of Bias 2 tool, as these symptoms are strong predictors of long-term prognosis in FEP. Disagreements were resolved through discussion or consultation with supervisors.	We identified 37 RCTs including 4599 participants. Participants' mean age was 25.8 years (SD 6.0), and 64.0% were men. We found evidence that psychological interventions (this component grouped all psychological treatment intended to treat, or ameliorate the consequences of, psychotic symptoms) are beneficial for reducing negative symptoms (iSMD −0.24, 95% CI −0.44 to −0.05, *p* = 0.014) at 3-month follow-up and may be associated with clinically relevant benefits in improving social functioning scores at 1-year follow-up (iSMD −0.52, 95% CI −1.05 to 0.01, *p* = 0.052). The addition of case management has a beneficial effect on reducing negative symptoms (iSMD −1.17, 95% CI −2.24 to −0.11, *p* = 0.030) and positive symptoms (iSMD −1.05, 95% CI −2.02 to −0.08, *p* = 0.033) at 1-year follow-up. Pharmacotherapy was present in all trial arms, making it impossible to examine the specific effects of this component.	In conclusion, this CNMA suggests that adding effective case management and psychological interventions to pharmacotherapy may modestly improve long-term symptoms and social functioning in early psychosis. Future work should aim to establish a gold-standard EIP service framework.	A few studies, sparse networks, small sample sizes, and reliance on indirect comparisons limit this review. Evidence certainty was very low, and publication bias or heterogeneity could not be fully assessed.

ARMS, At-Risk Mental State; CHEC, Consensus on Health Economic Criteria; CI, confidence interval; cNMA, component network meta-analysis; EIP, Early Intervention in Psychosis; EIS, Early Intervention Services; FEP, First-Episode Psychosis; HTA, Health Technology Assessment; iSMD, indirect standardized mean difference; PIN, Personal Identification Number; QALY, Quality-Adjusted Life-Year; RCT, randomized controlled trial; RoB 2, Risk of Bias 2; RR, risk ratio; SD, standard deviation; SMD, standardized mean difference; TAU, treatment as usual; UHR-T, Ultra-High Risk individuals who subsequently transitioned to psychosis.

### Data synthesis

The results were synthesized descriptively and presented in both narrative and tabular formats. The synthesis focused on summarizing and comparing findings across included systematic reviews and meta-analyses, identifying common themes, intervention characteristics, implementation factors, and evidence gaps related to early intervention mental health services. Findings were organized across the five pre-specified outcome domains of interest: effectiveness, accessibility, cost-effectiveness, patient satisfaction, and system-level outcomes.

Where available, information relating to outcome measures, synthesis approaches, heterogeneity, and sensitivity analyses reported within included meta-analyses was extracted and summarized. Given the rapid review framework and the objective of providing a timely synthesis of higher-level evidence, an independent formal methodological quality appraisal of included reviews using tools such as A Measurement Tool to Assess Systematic Reviews 2 (AMSTAR 2) or the Risk of Bias in Systematic Reviews (ROBIS) was not undertaken. However, risk-of-bias or appraisal information reported within included reviews was extracted where available, and the overall findings should therefore be interpreted cautiously.

## Results

**Figure 1:**
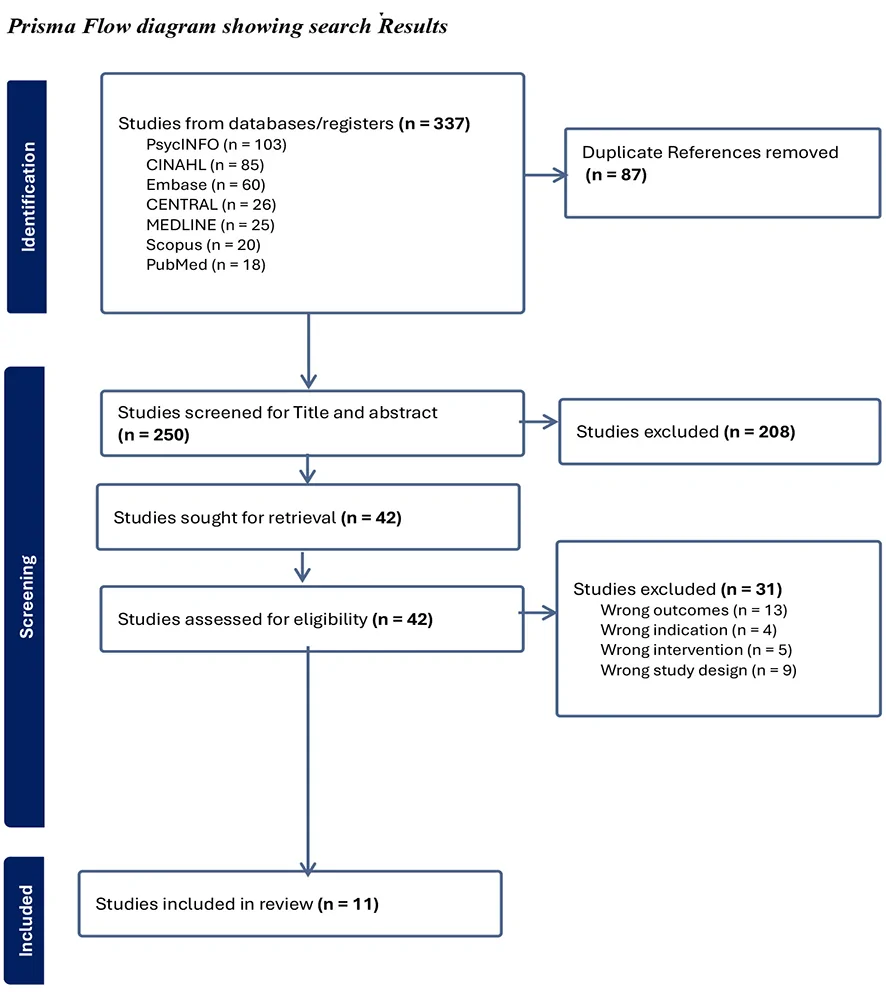
Prisma flow diagram showing results.

The study identified 337 studies from the databases searched. The Covidence software automatically screened and removed (87) studies as duplicates. The remaining items (250) were screened against the authors' eligibility criteria based solely on title and abstract, yielding 42 records for full-text screening. The remaining items were full text screened by the two reviewers, and 31 studies were excluded from the records. A total of **(11)** studies were then eligible for inclusion in this review. The study selection process is presented in Figure 1.

### Study characteristics

Eleven systematic reviews and meta-analyses met the inclusion criteria. The majority were conducted in high-income countries, with five originating in the United Kingdom, three in the United States, two in Australia, and one jointly from the United Kingdom and the Netherlands. Though the search strategy ranged from 2015 to 2025, the publication years of the included studies ranged from 2017 to 2024, reflecting growing global attention to the evaluation and implementation of early intervention services in mental health care.

Of the included reviews, three were quantitative systematic reviews with meta-analyses (38, 39), and one focused on economic evaluation (40). Six were systematic reviews with narrative syntheses (34, 41–45), and one was a meta-analysis of randomized controlled trials examining school-based psychological interventions (46). Collectively, these reviews synthesized evidence from 3 to 45 primary studies, encompassing total sample sizes ranging from 21 to 6,597 participants as per the reported. Study populations included individuals with or at risk of psychosis, children and youth presenting with acute or sub-clinical mental health conditions, and carers engaged in early psychosis programs.

### Scope of interventions

Interventions examined across the reviews were diverse. Core models included early intervention in psychosis (EIP) and early intervention services (EIS), which were compared against treatment as usual (23, 39); family and caregiver interventions (42); digital and smartphone-enabled interventions (34); and brief, crisis-focused approaches targeting children and adolescents (44). Additional studies explored predictors of service disengagement (41), transition pathways between child and adult mental health services (45), and barriers and facilitators to service access (43). Together, these reviews captured the breadth of early intervention modalities spanning clinical, psychosocial, technological, and system-level strategies.

### Clinical and service outcomes

Evidence from the included systematic reviews supports the effectiveness of early intervention approaches in improving clinical, functional, and service-level outcomes. In particular, a quantitative meta-analysis demonstrated that Early Intervention Services (EIS) and Early Intervention in Psychosis (EIP) programs significantly reduced treatment discontinuation, psychiatric hospitalization, and overall symptom severity compared with treatment as usual (23, 39).

Individuals entering services through at-risk mental state or high-risk clinics achieved outcomes comparable to or better than those entering directly with first-episode psychosis (38). School-based psychological interventions produced moderate reductions in depressive and anxiety symptoms among adolescents immediately after intervention, although benefits attenuated at longer follow-up (46).

Family-oriented programs improved caregiver knowledge, wellbeing, and participation in recovery, underscoring the importance of involving family systems in early psychosis care (42). Similarly, brief and crisis interventions for children and youth were associated with shorter hospital stays, reduced emergency department revisits, and improved short-term mental health outcomes (44).

### Service access, engagement, and disengagement

Across multiple reviews, service engagement and access emerged as critical determinants of treatment effectiveness (40, 42, 44). Disengagement rates from early psychosis services ranged from 12 to 53%, influenced by factors such as social isolation, limited family support, poor medication adherence, and substance misuse (41, 43, 45). Qualitative syntheses highlighted the pervasive impact of stigma, inadequate mental health literacy, and lack of supportive relationships, which collectively contributed to delayed help-seeking and prolonged duration of untreated psychosis (42, 43). Conversely, enabling factors such as family involvement, continuity of care, and early detection facilitated sustained engagement (41, 42, 44).

### Digital and technological innovations

Emerging evidence on digital interventions revealed high feasibility and user engagement among individuals with prodromal or early-phase psychosis (34). Smartphone-based applications have shown promise for symptom monitoring and integrating clinical feedback; however, most studies have been exploratory and lacked long-term follow-up (33).

None addressed integration with employment, education, or family support domains, suggesting an area for further innovation and evaluation (34).

### Economic and cost-effectiveness evidence

Economic analyses demonstrated that EIP models are cost-effective relative to standard care, offering better outcomes at lower or comparable costs (40). Savings were primarily attributed to reduced inpatient admissions, improved recovery trajectories, and enhanced functional outcomes (39). However, cost-effectiveness evidence was derived almost exclusively from high-income countries, limiting applicability to resource-constrained settings. Methodological limitations, including a lack of blinding and incomplete economic sensitivity analyses, further tempered certainty (39).

### Quality appraisal and risk of bias

Only two of the included studies explicitly reported their risk-of-bias assessment using standardized appraisal tools (39, 40). As no independent methodological quality appraisal was conducted in this rapid review, the overall certainty of the synthesized evidence should be interpreted cautiously. Formal appraisal tools, including Risk of Bias 2 (RoB 2) and the Consensus on Health Economic Criteria (CHEC) checklists, were employed in quantitative and economic evaluations respectively, (39, 40). The most common sources of bias included heterogeneity in study designs, absence of participant blinding, and incomplete reporting of secondary outcomes. Despite these limitations, the overall direction of findings remained consistent across studies, and the convergence of quantitative and qualitative evidence strengthened confidence in the main conclusions.

## Discussion

### Summary of the findings

This Rapid Review synthesized eleven systematic reviews and meta-analyses examining early intervention services across the mental health continuum. The evidence base provides moderate-to-high confidence that early intervention models, particularly those addressing early psychosis, family engagement, and youth crisis stabilization, are effective and cost-efficient in improving mental health outcomes. The literature reflects a steady global expansion of early intervention research, especially in high-income countries such as the United Kingdom, the United States, and Australia, where service frameworks are well-established and supported by policy and infrastructure.

The predominance of reviews on early psychosis underscores the maturity of this subfield, while more recent studies extend into youth crisis interventions, digital care innovations, and family-centered approaches (24, 35). These developments illustrate a transition from disorder-specific frameworks to more integrated, life-course-oriented approaches emphasizing prevention, early detection, acute stabilization, and relapse prevention (47). The integration of digital and family-centered components has enhanced feasibility, engagement, and service reach (34, 42); however, barriers such as stigma, low mental health literacy, and discontinuities in care persist as significant obstacles to timely access and sustained outcomes (43, 45).

Collectively, the findings reinforce the imperative for multi-component, person-centered, and system-integrated approaches to early intervention in mental health services. Strengthening implementation research, promoting cross-sector collaboration, and ensuring long-term evaluation will be critical to sustaining and scaling these gains globally.

### Types and characteristics of early intervention services

The identified reviews described a diverse range of intervention models. These included Early Intervention in Psychosis (EIP) and Early Intervention Services (EIS), which provide coordinated multidisciplinary care for individuals experiencing a first episode of psychosis or identified as being at high risk, with the aim of reducing the duration of untreated psychosis, improving recovery, and preventing hospitalization (23, 39). Care was typically delivered through specialized early psychosis teams comprising psychiatrists, psychologists, nurses, social workers, occupational therapists, and case managers who provided psychiatric treatment, psychological therapies, case management, psychoeducation, vocational support, and coordinated follow-up. Other models included family and caregiver interventions that promoted psychoeducation, communication, caregiver involvement, and treatment adherence while reducing caregiver burden (42); brief crisis stabilization programmes providing rapid assessment, short-term treatment, and timely transition to community services for children and youth experiencing mental health crises (44); and smartphone-based digital interventions supporting psychoeducation, real-time symptom monitoring, self-management, and communication with clinical teams (34). Service delivery models ranged from intensive case management and psychoeducation to blended digital–clinical approaches and transitional care frameworks bridging child and adult mental health services (45). This diversity indicates that early intervention is not a singular model but a continuum of services encompassing prevention, early detection, acute stabilization, and relapse prevention. Despite this progress, evidence from low- and middle-income countries remains limited, highlighting persistent inequities in the availability, design, and evaluation of early intervention programmes (40, 48, 49).

### Effectiveness of early intervention services

The evidence consistently supports the effectiveness of early intervention approaches in improving clinical, functional, and economic outcomes. Early intervention in psychosis models demonstrated significant reductions in hospitalization, treatment discontinuation, and symptom severity compared with treatment as usual, alongside improvements in employment and educational participation (23). Family interventions enhanced caregiver knowledge and involvement in recovery (42), while brief interventions for children and youth improved short-term stabilization and reduced emergency department revisits (44). Early intervention in psychosis services also produced better outcomes at lower or comparable costs (40). Smartphone-based digital interventions showed promise for symptom monitoring, self-management, and clinical communication, but the evidence remains largely exploratory and requires confirmation through larger randomized trials with longer follow-up (34). The findings of this rapid review are consistent with previous evidence demonstrating that coordinated, multidisciplinary early intervention improves clinical outcomes, reduces hospitalization, and enhances functional recovery (50, 51). Unlike previous reviews that focused on specific disorders or interventions, this review synthesizes evidence across multiple early intervention models, highlighting both the expanding scope of services and ongoing challenges in accessibility, continuity of care, and equity.

### Implementation factors: barriers and facilitators

Multiple contextual and service-level factors influence implementation success. Barriers include stigma, fragmented care pathways, lack of mental health literacy, inadequate workforce capacity, and limited integration across primary and secondary care (43). Facilitators include family engagement, supportive therapeutic relationships, clear referral pathways, and flexible service models (42, 43, 45). Disengagement rates from early psychosis services ranged between 12 and 53%, often driven by social isolation, substance misuse, or weak family support (41). These findings underscore the importance of designing services that are culturally responsive, family-centered, and built on principles of equity-oriented and person-centered care (52, 53). Digital and blended models offer potential to address accessibility challenges, yet their implementation is constrained by issues of digital literacy, privacy, and technological infrastructure (34, 54).

These findings are consistent with previous reviews highlighting stigma, poor mental health literacy, fragmented care pathways, and inadequate service integration as major barriers to early intervention, while emphasizing family involvement, coordinated multidisciplinary care, and person-centered service models as key facilitators (24, 55).

### Implications for policy and practice

The convergent evidence from this rapid review strongly supports early intervention mental health services as a high-impact, cost-effective cornerstone of modern mental health systems (24, 56). Policymakers should prioritize the integration of early intervention in psychosis (EIP) and broader early intervention services (EIS) as standard components of publicly funded mental health care rather than as time-limited pilots or specialty add-ons. The consistent reductions in hospitalization, symptom severity, and functional impairment demonstrate that early intervention delivers both clinical and system-level value.

Health system planners should invest in comprehensive, multidisciplinary EIS models that integrate medical, psychological, social, and vocational supports. The demonstrated benefits of family and caregiver interventions highlight the need for policies that formally embed family engagement as a funded and accountable component of early intervention pathways. This includes routine psychoeducation, caregiver support programs, and structured involvement in care planning.

The evidence that service disengagement undermines outcomes has important policy implications. Mental health systems must shift from passive referral-based models toward proactive engagement strategies that address social isolation, substance use, and fragmented care. Reducing stigma, strengthening mental health literacy, and ensuring continuity across child–adult service transitions should be regarded as system performance priorities rather than peripheral concerns. Targeted policy levers including performance indicators tied to engagement and early detection may help shorten duration of untreated psychosis and improve long-term recovery trajectories.

Emerging digital interventions offer important opportunities for scaling access, particularly in rural, remote, and equity-deserving populations. However, their current role remains adjunctive rather than substitutive. Policymakers should support the structured integration of digital tools into routine early intervention services with appropriate clinical governance, data security frameworks, and quality assurance mechanisms. Digital innovations should be aligned with workforce development strategies rather than viewed as replacements for in-person care.

Finally, the strong evidence for cost-effectiveness in high-income countries provides a compelling economic argument for sustained public investment. Ministries of Health and Treasury should view early intervention as a preventive health strategy with downstream returns on investment through reduced inpatient utilization, improved functional recovery, and enhanced workforce participation.

### Future directions

Despite substantial progress, several critical gaps remain that should guide future research, service development, and policy reform. First, there is a need for high-quality, long-term comparative studies assessing outcomes beyond 2 years, including sustained employment, educational attainment, social integration, and quality of life. Understanding the durability of early intervention effects across the life course is essential for informing long-term system planning. Second, the near-exclusive concentration of economic evidence in high-income countries highlights a major global evidence gap. Future studies must evaluate the feasibility, effectiveness, and cost-effectiveness of early intervention models in low- and middle-income countries, where the treatment gap and duration of untreated psychosis are often greatest. Adapted, stepped-care, and task-shared EIS models may be particularly relevant in these settings.

Third, digital and hybrid care models require more rigorous evaluation. Future trials should move beyond feasibility and short-term symptom monitoring to examine whether digital interventions can meaningfully enhance engagement, reduce relapse, support vocational recovery, and strengthen family involvement. Integration across clinical, educational, employment, and social domains remains an under-researched but potentially transformative area. Fourth, persistent disengagement and barriers to access underscore the need for implementation science approaches that address real-world complexity. Future research should focus on culturally responsive care models, youth-friendly engagement strategies, and structural interventions that reduce stigma and administrative fragmentation across services.

Finally, future policy development should emphasize whole-system integration, linking early intervention services with primary care, emergency departments, schools, and community-based supports. Strengthening data infrastructure to enable real-time monitoring of outcomes, equity of access, and service performance will be essential to guide continuous quality improvement. Embedding standardized outcome measurement and evaluation frameworks, such as those proposed in PRISMA and WHO action plans, will strengthen accountability and guide cross-system learning (53, 56, 57).

### Strengths and limitations

The strength of this review lies in its broad scope and structured synthesis across multiple domains of early intervention. By consolidating evidence from both quantitative and qualitative reviews, this work provides a panoramic understanding of effectiveness, accessibility, and implementation barriers. However, limitations include reliance on secondary data, heterogeneity in definitions and outcomes, and the concentration of evidence in high-income contexts. Also, the search criteria were limited only to selected databases and English Language publications and may have excluded other studies. Furthermore, as no independent methodological quality appraisal was conducted in this rapid review, the overall certainty of the synthesized evidence should be interpreted cautiously. Future research should include low-resource and culturally diverse settings to enhance global relevance.

## Conclusions

This rapid review of systematic reviews and meta-analyses provides compelling, high-level evidence that early intervention mental health services are clinically effective, operationally efficient, and economically sound. Across diverse models including early intervention in psychosis, youth-focused crisis services, family-based interventions, and emerging digital tools. Early intervention consistently outperforms treatment as usual in reducing symptom severity, psychiatric hospitalization, treatment discontinuation, and functional impairment. These benefits extend beyond clinical recovery to include improved social and occupational functioning, reinforcing the role of early intervention as a foundational strategy for long-term mental health outcomes.

Importantly, this review highlights that the effectiveness of early intervention is strongly contingent upon timely access and sustained engagement. High rates of disengagement, stigma, and system fragmentation continue to undermine potential benefits, emphasizing that early intervention must be embedded within responsive, youth and family friendly systems of care. While digital innovations show promise for enhancing monitoring and access, their current evidence base remains preliminary, underscoring the need for cautious integration alongside robust clinical governance.

From a health systems perspective, the available economic evidence supports early intervention as a cost-effective investment, primarily through reductions in inpatient utilization and improved recovery trajectories. However, the concentration of economic evaluations in high-income countries limits generalizability and reinforces the urgency for implementation and economic research in low- and middle-income settings, where the treatment gap remains greatest.

Overall, the findings of this review affirm early intervention as one of the strongest evidence-based strategies for transforming population mental health outcomes. The next phase of impact will depend not on whether early intervention works, but on how effectively it is scaled, equitably implemented, digitally enabled, and sustained across diverse health systems. Aligning policy, workforce, funding, and community engagement around early intervention offers a powerful opportunity to shift mental health care from crisis-driven responses toward proactive, recovery-oriented systems of care.

## Data Availability

The original contributions presented in the study are included in the article/Supplementary material, further inquiries can be directed to the corresponding author.
