# Supplementary material for: Early interventions in mental health services: a rapid review of systematic review
Source: Front Public Health. 2026 Sep 3;14:1782381. doi: 10.3389/fpubh.2026.1782381 (PMC13581798; doi:10.3389/fpubh.2026.1782381)
Supplement: Supplementary file 1 [file Supplementary_File_1.docx]

**Supplementary Appendix 1. Database Search Strategies**

Searches were conducted between June 15 and June 30, 2025. Controlled vocabulary terms (e.g., MeSH, PsycINFO Subject Headings, and CINAHL Headings) were combined with free-text keywords using Boolean operators. Searches were limited to English-language publications, human populations, and systematic reviews or meta-analyses published between January 2015 and June 2025. Database-specific syntax and indexing terms were adapted as appropriate. The final number of records retrieved from each database is reported with the corresponding search strategy, with **337** records identified across all databases before deduplication.

**MEDLINE (Ovid) (n = 25)**

1. exp Early Intervention/
2. exp Crisis Intervention/
3. (early intervention* OR early treatment* OR early management OR preventive care OR early clinical intervention* OR early-phase therap* OR pre-clinical intervention* OR early detection).ti,ab,kf.
4. 1 OR 2 OR 3

5. exp Mental Health Services/
6. exp Community Mental Health Services/
7. exp Psychiatric Services/
8. (mental health service* OR mental health care OR mental health care delivery OR mental health intervention* OR psychiatric service* OR behavioural health service* OR behavioral health service* OR psychological care OR psychosocial support service* OR therapeutic mental health service* OR mental health treatment program* OR mental health support system* OR mental healthcare system* OR MHPSS).ti,ab,kf.
9. 5 OR 6 OR 7 OR 8

10. Meta-Analysis as Topic/
11. Meta-Analysis.pt.
12. Systematic Review.pt.
13. (systematic review* OR meta-analy* OR review of reviews).ti,ab,kf.
14. 10 OR 11 OR 12 OR 13
**15. 4 AND 9 AND 14**

**EMBASE Search Strategy (n = 60)**

('early intervention'/exp OR 'early intervention':ti,ab,kw OR 'early treatment':ti,ab,kw OR 'early management':ti,ab,kw OR 'preventive care':ti,ab,kw OR 'early clinical intervention':ti,ab,kw OR 'early phase therap*':ti,ab,kw OR 'pre-clinical intervention*':ti,ab,kw OR 'early detection':ti,ab,kw OR 'crisis intervention'/exp OR 'crisis intervention*':ti,ab,kw)

AND

('mental health service'/exp OR 'psychiatric service'/exp OR 'community mental health service'/exp OR 'mental health service*':ti,ab,kw OR 'mental health care':ti,ab,kw OR 'mental health care delivery':ti,ab,kw OR 'mental health intervention*':ti,ab,kw OR 'behavioral health service*':ti,ab,kw OR 'behavioural health service*':ti,ab,kw OR 'psychological care':ti,ab,kw OR 'psychosocial support service*':ti,ab,kw OR 'therapeutic mental health service*':ti,ab,kw OR 'mental health treatment program*':ti,ab,kw OR 'mental health support system*':ti,ab,kw OR 'mental healthcare system*':ti,ab,kw OR 'mental health and psychosocial services':ti,ab,kw OR MHPSS:ti,ab,kw)

AND

('systematic review'/exp OR 'meta analysis'/exp OR 'systematic review':ti,ab,kw OR 'meta-analysis':ti,ab,kw OR 'meta analysis':ti,ab,kw OR 'review of reviews':ti,ab,kw)

**CINAHL (EBSCOhost) (**n = 85)

(MH "Early Intervention")

(MH "Crisis Intervention")

TI ("early intervention*" OR "early treatment*" OR "early management" OR "preventive care" OR "early clinical intervention*" OR "early phase therap*" OR "pre-clinical intervention*" OR "early detection") OR AB ("early intervention*" OR "early treatment*" OR "early management" OR "preventive care" OR "early clinical intervention*" OR "early phase therap*" OR "pre-clinical intervention*" OR "early detection")

S1 OR S2 OR S3

(MH "Mental Health Services+")

(MH "Community Mental Health Services+")

(MH "Psychiatric Services+")

TI ("mental health service*" OR "mental health care" OR "mental health care delivery" OR "mental health intervention*" OR "psychiatric service*" OR "behavioral health service*" OR "behavioural health service*" OR "psychological care" OR "psychosocial support service*" OR "therapeutic mental health service*" OR "mental health treatment program*" OR "mental health support system*" OR "mental healthcare system*" OR MHPSS) OR AB ("mental health service*" OR "mental health care" OR "mental health care delivery" OR "mental health intervention*" OR "psychiatric service*" OR "behavioral health service*" OR "behavioural health service*" OR "psychological care" OR "psychosocial support service*" OR "therapeutic mental health service*" OR "mental health treatment program*" OR "mental health support system*" OR "mental healthcare system*" OR MHPSS)

S5 OR S6 OR S7 OR S8

(MH "Systematic Review")

(MH "Meta Analysis")

TI ("systematic review*" OR "meta-analy*" OR "review of reviews") OR AB ("systematic review*" OR "meta-analy*" OR "review of reviews")

S10 OR S11 OR S12

S4 AND S9 AND S13

**PubMed (n = 18)**

("early intervention"[Title/Abstract]

OR "early treatment"[Title/Abstract]

OR "early management"[Title/Abstract]

OR "preventive care"[Title/Abstract]

OR "early clinical intervention"[Title/Abstract]

OR "early phase therapy"[Title/Abstract]

OR "pre-clinical intervention"[Title/Abstract]

OR "early detection"[Title/Abstract]

OR "crisis intervention"[Title/Abstract])

AND

("mental health services"[Title/Abstract]

OR "psychiatric services"[Title/Abstract]

OR "mental health care"[Title/Abstract]

OR "mental health care delivery"[Title/Abstract]

OR "mental health intervention*"[Title/Abstract]

OR "behavioral health services"[Title/Abstract]

OR "behavioural health services"[Title/Abstract]

OR "psychological care"[Title/Abstract]

OR "psychosocial support services"[Title/Abstract]

OR "mental healthcare systems"[Title/Abstract]

OR MHPSS[Title/Abstract])

AND

(systematic review[Publication Type]

OR meta-analysis[Publication Type]

OR "systematic review"[Title/Abstract]

OR "meta-analysis"[Title/Abstract]

OR "review of reviews"[Title/Abstract])

**PsycINFO (Ovid) (n = 103)**

1. exp Early Intervention/

2. exp Crisis Intervention/

3. (early intervention* OR early treatment* OR early management OR preventive care OR early clinical intervention* OR early-phase therap* OR pre-clinical intervention* OR early detection).ti,ab,id.

4. 1 OR 2 OR 3

5. exp Mental Health Services/

6. exp Community Mental Health Services/

7. exp Psychiatric Services/

8. (mental health service* OR mental health care OR psychiatric service* OR behavioural health service* OR behavioral health service* OR psychological care OR psychosocial support service* OR mental healthcare system* OR MHPSS).ti,ab,id.

9. 5 OR 6 OR 7 OR 8

10. Meta Analysis/

11. Systematic Review/

12. (systematic review* OR meta-analy* OR review of reviews).ti,ab,id.

13. 10 OR 11 OR 12

**14. 4 AND 9 AND 13**

**Cochrane Library / CENTRAL (n = 26)**

("early intervention"

OR "early treatment"

OR "early management"

OR "preventive care"

OR "early clinical intervention"

OR "early phase therapy"

OR "pre-clinical intervention"

OR "early detection"

OR "crisis intervention")

AND

("mental health services"

OR "psychiatric services"

OR "mental health care"

OR "mental health interventions"

OR "behavioral health services"

OR "behavioural health services"

OR "psychological care"

OR "psychosocial support services"

OR "mental healthcare systems"

OR MHPSS)

AND

("systematic review"

OR "meta-analysis"

OR "review of reviews")

**Scopus (n = 20)**

("early intervention"

OR "early treatment"

OR "early management"

OR "preventive care"

OR "early clinical intervention"

OR "early phase therapy"

OR "pre-clinical intervention"

OR "early detection"

OR "crisis intervention")

AND

("mental health services"

OR "psychiatric services"

OR "mental health care"

OR "mental health care delivery"

OR "mental health interventions"

OR "behavioral health services"

OR "behavioural health services"

OR "psychological care"

OR "psychosocial support services"

OR "mental healthcare systems"

OR MHPSS)

AND

("systematic review"

OR "meta-analysis"

OR "review of reviews")
